# Atorvastatin Inhibits Inflammatory Response, Attenuates Lipid Deposition, and Improves the Stability of Vulnerable Atherosclerotic Plaques by Modulating Autophagy

**DOI:** 10.3389/fphar.2018.00438

**Published:** 2018-05-03

**Authors:** Shi Peng, Long-Wei Xu, Xin-Yu Che, Qing-Qing Xiao, Jun Pu, Qin Shao, Ben He

**Affiliations:** Department of Cardiology, Renji Hospital, School of Medicine, Shanghai Jiao Tong University, Shanghai, China

**Keywords:** atherosclerosis, atorvastatin, autophagy, inflammatory response, vulnerable plaque stability

## Abstract

Atherosclerosis is a chronic disease comprising intima malfunction and arterial inflammation. Recent studies have demonstrated that autophagy could inhibit inflammatory response in atherosclerosis and exert subsequent atheroprotective effects. Our previous study also demonstrated the role of autophagy in the inhibition of inflammation by atorvastatin *in vitro*. Therefore, in the present study, we aimed to determine whether atorvastatin could upregulate autophagy to inhibit inflammatory cytokines secretion, lipid accumulation, and improve vulnerable plaque stability, both *in vitro* and *in vivo*. First, we established a vulnerable atherosclerotic plaque mouse model through partial ligation of left common carotid artery and left renal artery to explore the effect of atorvastatin on vulnerable plaques. The results showed that atorvastatin could enhance the stability of vulnerable atherosclerotic plaques and reduce the lesion area in the aorta. Atorvastatin could also inhibit NLRP3 inflammasome activation and inflammatory cytokines, such as IL-1β, TNF-α, and IL-18 secretion *in vivo*. Atorvastatin treatment upregulated the expression of autophagy-related protein microtubule-associated protein light chain (LC3B) and downregulated the expression of SQSTM1/p62, which suggested that autophagy was activated in vulnerable plaques. Transmission electron microscopy further demonstrated the atorvastatin-induced increase in autophagy activity in vulnerable atherosclerotic plaques. We employed oxidized low-density lipoprotein (ox-LDL) to stimulate RAW264.7 cells with atorvastatin, which showed that atorvastatin could attenuate lipid deposition, ameliorate inflammation, inhibit NLRP3 inflammasome activation, and enhance autophagy *in vitro*. All these beneficial effects were abolished by 3-methyladenine treatment, an autophagy inhibitor. Atorvastatin also significantly inhibited the phosphorylation of mTOR, which strongly suggested the involvement of the mTOR pathway. Our study proposed a new role for atorvastatin as an autophagy inducer to exert anti-inflammatory and atheroprotective effects, to stabilize vulnerable atherosclerotic plaques.

## Introduction

Atherosclerosis is a chronic inflammatory vascular disease that begins with lipid deposition under the endothelium (Crea and Libby, 2017). Vascular inflammation aggravates the development of atherosclerosis and deteriorate atherosclerosis plaques; as a result, vulnerable atherosclerotic plaques tend to rupture (Kirii et al., 2003; de Nooijer et al., 2004; Kavurma et al., 2017). Libby reported that canakinumab, a monoclonal antibody targeting interleukin (IL)-1β, can reduce the risk of cardiovascular events in a subset of patients with a history of myocardial infarction and a high level of C-reactive protein (Ridker et al., 2017). All of which directly proved that anti-inflammatory therapy could inhibit the development of cardiovascular disease.

Statins are inhibitors of 3-hydroxyl-3-methylglutaryl coenzyme A (HMG-CoA) reductase, a key enzyme in the synthesis of cholesterol (Gotto, 2003). Currently, statins are widely used to control cholesterol levels and have achieved very good effects in lowering the incidence of cardiovascular events among patients. Atorvastatin is a statin that has become a routine treatment for patients with hypercholesterolemia and atherosclerosis. Intriguingly, in addition to its lipid-lowering ability, atorvastatin can also produce anti-inflammatory effects (Shao et al., 2012). Furthermore, atorvastatin is associated with increased autophagy in prostate PC3 cells (Parikh et al., 2010; Toepfer et al., 2011; Gao et al., 2016). Our previous study showed that pretreatment with atorvastatin could significantly ameliorate the secretion of IL-1β and tumor necrosis factor alpha (TNF-α) in macrophages induced by lipopolysaccharide (LPS) (Han et al., 2017). However, the role played by atorvastatin in advanced atherosclerotic plaques and its potential mechanism have not been determined.

Autophagy is a highly conserved biological process that begins with a phagopore, which contains isolated cargoes that need to be degraded. The phagophores elongate and then form autophagosomes. Finally, lysosomes are fused to form autolysosomes (De Meyer et al., 2015). Eventually, aging organelles or misfolded proteins are degraded by hydrolytic enzymes and are recycled or used to provide energy. The whole process is termed autophagy flux (Levine et al., 2011).

Autophagy is tightly associated with the vulnerability of atherosclerotic plaques (Zhai et al., 2014; Magne et al., 2015), and loss of the autophagy protein Atg 16L1 exacerbates the inflammatory response (Saitoh et al., 2008). Moreover, autophagy dysfunction has been shown to promote the development of atherosclerosis. Accumulating evidence has revealed that upregulation of autophagy can modulate the progression of atherosclerosis and decrease the vulnerability of atherosclerotic plaques (Leng et al., 2016).

Inflammasomes are a class of multiprotein complexes that include NLR family pyrin domain containing 3 (NLRP3) inflammasomes and absent in melanoma 2 (AIM2) inflammasomes, among which NLRP3 inflammasomes have attracted the most attention, which contain NLRP3, apoptosis-associated speck-like protein containing a CARD (ASC), and pro-caspase-1 (Mariathasan et al., 2006; Shi et al., 2012). Inflammasomes can be triggered by infection or stress, and once activated, caspase-1 is responsible for processing the precursors of IL-1β and IL-18 into their mature forms, which are secreted into the extracellular environment (Man et al., 2014).

A growing body of evidence has demonstrated that NLRP3 inflammasomes play a pivotal role in inflammation and cholesterol metabolism (Rajamaki et al., 2010). Activated NLRP3 inflammasomes exacerbate macrophage lipid deposition and atherosclerosis (Li et al., 2014). However, whether the role of atorvastatin in vulnerable atherosclerotic plaques acts via modulating autophagy and inflammasome inhibition, and the signaling pathways involved, are not fully understood.

In the present study, we investigated the precise role of atorvastatin in autophagy, foam cell formation, and inflammation in macrophages with oxidized low-density lipoprotein (ox-LDL) stimuli and whether it could stabilize vulnerable atherosclerotic plaques by enhancing autophagy.

## Materials and Methods

### Animals

Male ApoE^-/-^ mice (8 weeks old, 22–26 g) on a C57BL/6 background (*n* = 40) were purchased from the Shanghai Biomodel Organism Science and Technology Development Co., Ltd. (Shanghai, China) and housed in a specific pathogen free (SPF) environment at a stable temperature (18–22°C) and humidity level (50–70%) and under a 12 h dark/12 h light cycle in the Animal Care Facility of Shanghai Jiao Tong University, School of Medicine. During the whole experimental period, the mice had free access to water and food. The ApoE^-/-^ mice were provided with a high-fat-diet (HFD) (Research Diets, Inc., New Brunswick, NJ, United States) containing 1.25% cholesterol, 20% fat, and 0.5% sodium cholate. All animal work was carried out according to guidelines for the care and use of laboratory animals of the Shanghai Jiao Tong University School of Medicine.

### Animal Model

The animal experiment grouping is described in detail in Supplementary Figure 1. In brief, mice were divided into four groups: HFD (mice without surgery), vehicle (saline solution), Ator 10 (atorvastatin 10 mg/kg/day), and Ator 20 (atorvastatin 10 mg/kg/day). At the age of 8 weeks, ligation surgery was performed, as previously described (Sullivan, 2002; Korshunov and Berk, 2003; Nam et al., 2009; Jin et al., 2012). Briefly, the ApoE knockout mice (*n* = 40) were anesthetized by intraperitoneal injection of 3.5% chloral hydrate and fixed for surgery. The left common carotid artery and its branches were exposed by blunt dissection, being careful not to damage the vagus nerve and carotid sinus. The occipital artery and internal carotid artery ligation were implemented and ligation of external carotid artery was performed after the superior thyroid artery was separated from the external carotid artery. The wound was stitched using a 6–0 suture and the animals were resuscitated on a thermostat plate until they were completely awake, and then returned to the cage. Partial ligation of the left renal artery was performed as described previously (Sullivan, 2002; Korshunov and Berk, 2003; Nam et al., 2009; Jin et al., 2012; Nie et al., 2014). Atorvastatin (Pfizer Ltd., New York, NY, United States) was dissolved in saline solution and administrated to the mice by gavage at a dose of 10 mg/kg/day or 20 mg/kg/day after surgery for 8 weeks. The mice were sacrificed by cervical dislocation 8 weeks after the partial ligation surgery of left renal artery.

### Antibodies

Rabbit polyclonal anti-microtubule-associated proteins 1A/1B light chain 3A (LC3), anti-sequestosome 1 (SQSTM1)/p62, anti-NLRP3, anti-phospho-mechanistic target of rapamycin kinase (mTOR), anti-mTOR, and anti-glyceraldehyde-3-phosphate dehydrogenase (GAPDH) were purchased from Cell Signaling Technology Inc. (Danvers, MA, United States). Rabbit monoclonal anti-LC3B, anti-SQSTM1/p62, anti-IL-1β, and Goat polyclonal anti-NLRP3 were purchased from Abcam (Cambridge, MA, United States).

### En Face Oil Red O Staining

Aortas from the aortic arch to the iliac artery were dissected under a dissecting microscope and soaked in 4% paraformaldehyde overnight. The aortas were opened longitudinally and stained with 0.5% Oil Red O solution (Sigma-Aldrich) and photographed on a black background. The images were analyzed using Image Pro-Plus 6.0 (Media Cybernetics, Rockville, MD, United States).

### Enzyme-Linked Immunosorbent Assay (ELISA)

The level of inflammatory cytokines in the serum and cell culture supernatant were measured using an enzyme-linked immunosorbent assay (ELISA) kit (eBioscience, San Diego, CA, United States) according to the manufacturer’s instructions. Blood samples were centrifuged at 2,000 rpm for 15 min at 4°C and cell culture medium was centrifuged at 2,500 rpm for 10 min at 4°C. Both supernatants were stored at -80°C.

### Immunofluorescence Staining

The left common carotid artery was washed with saline solution and embedded in optimal cutting temperature compound (Sakura Finetek USA, Inc., Torrance, CA, United States). Serial cryosections (5-μm) were obtained using a cryotome (FSE, Thermo Scientific, Rockford, IL, United States). The sections were thawed at room temperature for 30 min, followed by fixation with pre-cooled methanol for 10 min at room temperature. The sections were rehydrated with phosphate-buffered saline (PBS) for 10 min and then washed in PBS twice for 5 min each. After blocking with 5% bovine serum albumin (Roche) for 30 min at room temperature, the sections were incubated with a rabbit polyclonal antibody against IL-1β (ab9722, Abcam) or a goat polyclonal antibody against NLRP3 (ab4207, Abcam) overnight at 4°C. The sections were then washed with PBS three times and incubated with an Alexa Fluor488 donkey anti-rabbit secondary antibody (A-21206, Life Technologies, Grand Island, NY, United States) or an Alexa Fluor594 donkey anti-goat secondary antibody (ab150136, Abcam) for 60 min at room temperature. To detect the expression of autophagy markers in vulnerable atherosclerotic plaques, frozen sections were incubated with a rabbit monoclonal antibody against LC3B (ab192890, Abcam) or a rabbit monoclonal antibody against SQSTM1/p62 (ab109012, Abcam) overnight at 4°C and incubated with an Alexa Fluor488 donkey anti-rabbit secondary antibody (A-21206, Life Technologies). After washing three times with PBS, the sections were mounted with antifade mountant with DAPI (P36965, ProLong Diamond Antifade Mountant, Life Technologies, Grand Island, NY, United States) for 15 min at room temperature. Fluorescence images were captured under a confocal microscope (Zeiss710, ZEISS, Germany).

RAW264.7 macrophage cells that underwent different treatments were rinsed with PBS and fixed with 4% paraformaldehyde for 10 min at room temperature, followed by permeabilization with 0.1% Triton X-100 for 10 min. 5% bovine serum albumin was used to block non-specific staining and the coverslips were incubated with anti-NLRP3, anti-IL-1β, anti-LC3B, or anti-SQSTM1/p62 antibodies. The subsequent steps were the same as those detailed above.

### Transmission Electron Microscopy (TEM)

The electron microscopy sample as prepared as is described previously (Rosenfeld et al., 2000; Perrotta, 2013). In brief, the left common carotid artery was fixed with 2.5% glutaraldehyde, and then postfixed with 3% osmium tetroxide (OsO_4_) for 2 h. The specimen was dehydrated in a graded series of ethanol, and embedded in Epon resin. For TEM study, ultrathin sections (40–60 nm) were obtained and observed using a PHILIPS CM-120 electron microscope.

### Cell Culture

Murine macrophage RAW264.7 cells were purchased from the American Type Culture Collection (ATCC) and cultured in Dulbecco’s Modified Eagle Medium (DMEM, Life Technologies) supplemented with 10% fetal bovine serum (Life Technologies). For experiments, RAW264.7 cells were treated with atorvastatin (100 μM) and ox-LDL (50 or 100 μg/ml, Yiyuan Biotechnologies, Guangzhou, China) either alone or in combination. Cells were pretreated with 3-MA (3-methyladenine; 5 mM) for 1 h to impede autophagy activity. The rapamycin dosage was 10 nM. After different treatments, cells were collected and subjected to subsequent experiments.

### Oil Red O Staining

Cultured RAW264.7 cells were plated in six-well plates at a density of 2 × 10^5^ cells/well and incubated with or without atorvastatin and ox-LDL for 24 h. 3-MA was used to block autophagy induced by atorvastatin. Cells were washed with PBS twice and then fixed with 10% formalin for 10 min. The cells were then washed with PBS twice and stained with Oil Red O working solution for 30 min at room temperature. Cells were washed with PBS until the background was clean and the photographed under a microscope (Leica DM3000B, Germany).

### Western Blotting

The protein levels of LC3, p62, NLRP3 in atherosclerotic plaques of ApoE^-/-^ mice and LC3, p62, NLRP3, mTOR/p-mTOR in RAW264.7 cells were analyzed using western blotting. Western blotting analyses were performed as follows. Total proteins were extracted from RAW264.7 cells seeded on a 6-well plate by rinsing with ice-cold PBS or from atherosclerotic plaques in the aortas of the ApoE^-/-^ mice, and lysed in lysis buffer (Roche) containing protease and phosphatase inhibitors (78442, Thermo Fisher Scientific, United States). Lysates were then collected after centrifugation. The protein concentration was measured using a Bicinchoninic acid Protein Assay Reagent (Pierce, United States). The proteins was separated by 7.5–12.5% SDS-polyacrylamide gel electrophoresis and transferred to polyvinylidene difluoride membranes (Millipore). After blocking with 5% non-fat milk for 1 h at room temperature, the membranes were incubated with different primary antibodies overnight at 4°C. The membranes were washed three times with TBST and incubated with horseradish peroxidase-conjugated secondary antibodies (Jackson ImmunoResearch Laboratories, Inc., West Grove, PA, United States) for 1 h at room temperature. The bands were detected using an ImageQuant LAS 4000 Imager, and gray-scale value analysis was performed using the Gel-Pro analyzer.

### Terminal Deoxynucleotidyl Transferase-Mediated dUTP-Biotin Nick End Labeling (TUNEL) Assay

See Supplementary Materials.

### Statistics

The data are presented as the mean ± standard error of the mean (SEM) and were calculated using GraphPad Prism 5.0 (GraphPad Software, San Diego, CA, United States). All the cell experiments were independently repeated at least three times. An unpaired Student’s *t*-test was used to compare the data in different groups. The significance of the differences was assessed by one-way analysis of variance (ANOVA) followed by a Newman–Keuls test. A *P*-value < 0.05 was considered statistically significant.

## Results

### Atorvastatin Treatment Enhanced the Stability of Vulnerable Atherosclerotic Plaques and Reduced Aortic Plaque Area

**FIGURE 1:**
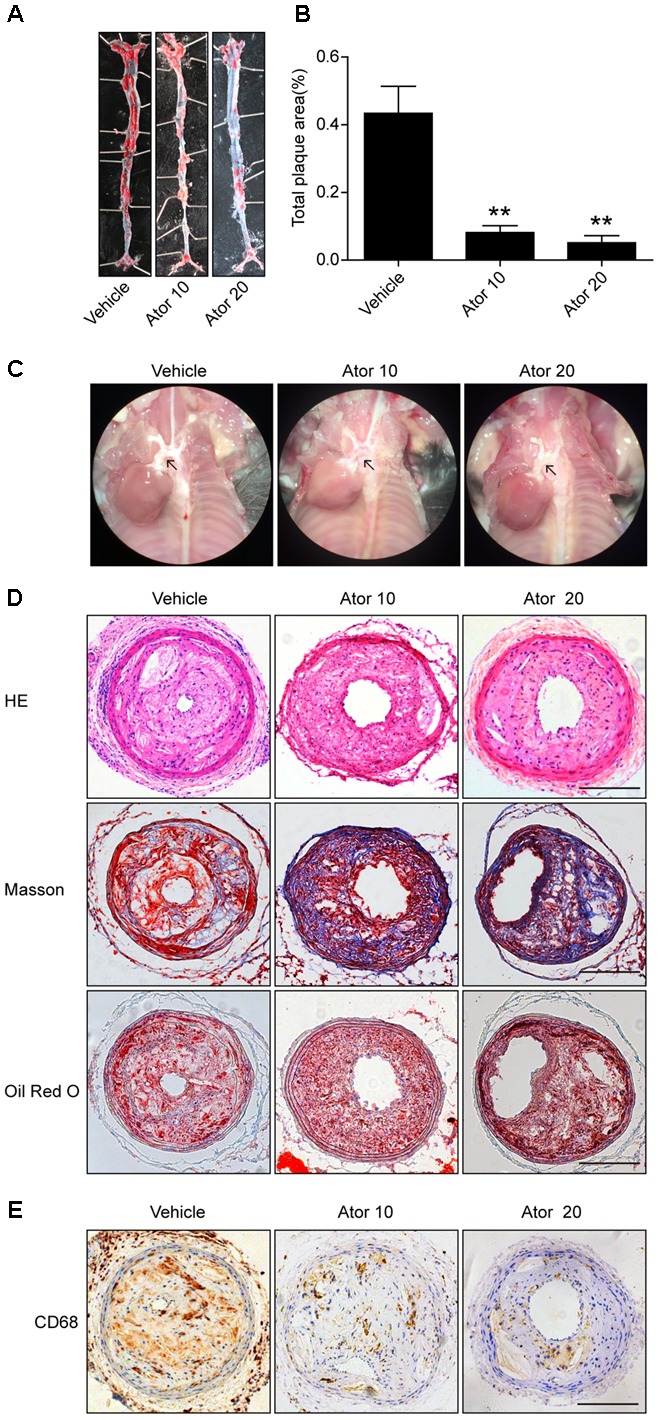
Atorvastatin markedly improved the stability of vulnerable atherosclerotic plaques in ApoE^-/-^ mice. **(A)** Representative images of the en face aorta Oil Red O staining plaque burden (red) from vehicle-treated and atorvastatin-treated mice. **(B)** Gross morphology and quantification of plaque areas in entire aortas, *n* = 3. **(C)** Anatomical view of the aortic arch from different groups under a dissecting microscope; black arrowheads represent atherosclerotic lesions in the aortic arch. **(D)** Vulnerable atherosclerotic plaques in the left common carotid artery were collected and sectioned. These sections were stained with hematoxylin and eosin (HE), Masson stain, and Oil Red O, scale bar = 200 μm. **(E)** Representative immunohistochemical staining of CD68 for macrophages, scale bars = 200 μm. Data are presented as mean ± SEM. ^∗^*P* < 0.05 vs. the vehicle group, ^∗∗^*P* < 0.01 vs. the vehicle group. Vehicle, mice were given saline solution alone. Ator 10, atorvastatin at 10 mg/kg/day. Ator 20, atorvastatin at 20 mg/kg/day.

To explore the effect of atorvastatin on the development of atherosclerosis, we established a vulnerable atherosclerotic plaque animal model. At the age of 16 weeks, the ApoE^-/-^ knockout mice were euthanized and the left common carotid artery and the aorta were excised. The carotid artery was paraffin-embedded or optimal cutting temperature-embedded and made into frozen sections or paraffin sections. The en face area of aorta was stained with Oil Red O. As shown in **Figures 1A–C**, atorvastatin significantly reduced the plaque area in the whole aorta and aortic arch compared with the vehicle group (*p* < 0.05). Furthermore, hematoxylin and eosin staining, Masson staining, and Oil Red O staining of carotid plaques also showed that atorvastatin could increase the amount of collagen and decrease lipid deposition in the vulnerable atherosclerotic plaques (**Figure 1D**). Thus, atorvastatin could improve plaque stability. However, atorvastatin did not influence the area of the vulnerable plaques (**Figure 1D**), which agreed with our previous research (Nie et al., 2014). The expression of CD68 is tightly correlated with the progression and rupture of vulnerable plaques (Woollard and Geissmann, 2010). Atorvastatin substantially alleviated CD68 expression in the plaques (**Figure 1E**), which suggested decreased macrophage infiltration and the protective effect of atorvastatin on the rupture of vulnerable plaques.

### Atorvastatin Effectively Suppressed Inflammation

**FIGURE 2:**
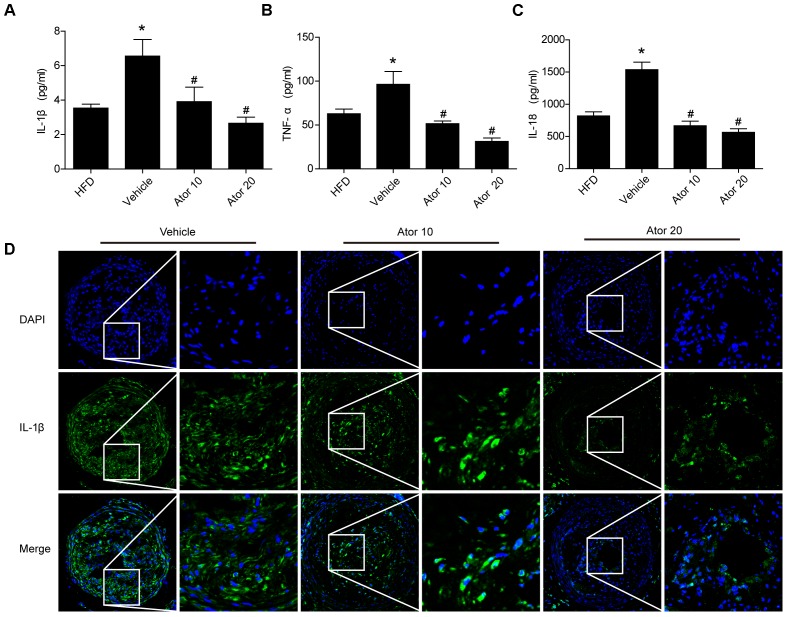
Atorvastatin inhibits the serum inflammatory response in ApoE^-/-^ mice. Blood was collected 8 weeks after saline or atorvastatin administration. **(A–C)** Histograms showed that the secretion levels of IL-1β, TNF-α, and IL-18 were increased markedly in the vehicle-treated group compared with those in the high-fat diet (HFD) group, whereas, atorvastatin significantly decreased the levels of IL-1β, TNF-α, and IL-18 compared with those in the vehicle group, *n* = 3. Data represent the mean ± SEM of three ELISA experiments. **(D)** The expression of IL-1β in vulnerable plaques was also assessed by immunofluorescence staining. ^∗^*P* < 0.05 vs. the HFD group alone, ^#^*P* < 0.05 vs. the vehicle group alone.

To illustrate the effect of atorvastatin on atherosclerotic inflammation, inflammatory cytokines, including IL-1β, TNF-α, and IL-18, in the blood serum of the ApoE^-/-^ knockout mice were detected using an ELISA assay. As shown in **Figures 2A–C**, mice with advanced atherosclerotic lesions had higher levels of serum inflammatory cytokines than mice fed on a high-fat diet. Atorvastatin significantly reduced the levels of IL-1β, TNF-α, and IL-18 compared with those in the saline group (*p* < 0.05); however, there was no significant difference in the effects between the two concentrations of atorvastatin. Immunofluorescence staining of IL-1β was then carried out, which showed that atorvastatin treatment significantly decreased the level of IL-1β in the vulnerable atherosclerotic plaques (**Figure 2D**). Therefore, atorvastatin could inhibit the inflammatory response in atherosclerotic mice.

### Atorvastatin Significantly Inhibited the Activation of NLRP3 Inflammasomes

**FIGURE 3:**
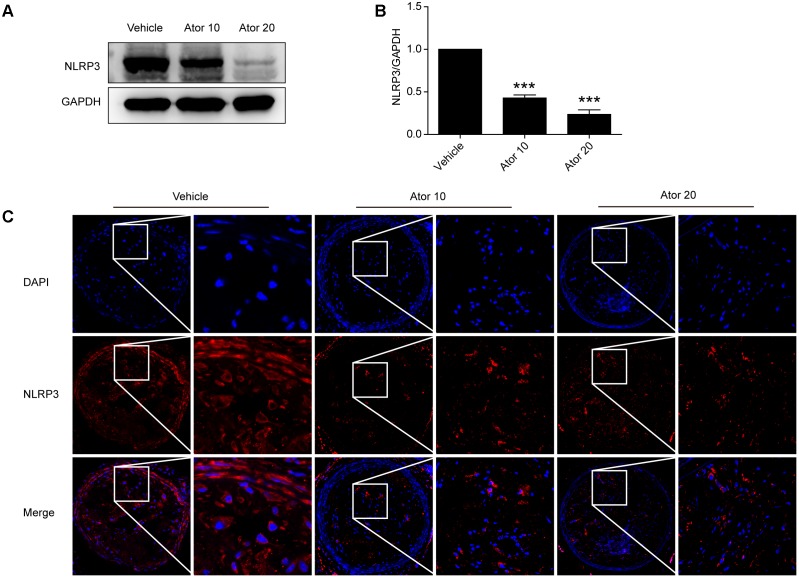
Effects of atorvastatin on inflammasome activation. **(A)** Mouse aortas were collected and western blotting analysis was carried out to detect the abundance of the NLRP3 inflammasome in aorta tissues. The GAPDH level served as the control. **(B)** Histogram showing the quantitative analysis of NLRP3 and GAPDH levels, *n* = 3. **(C)** Immunofluorescence staining of NLRP3 in vulnerable atherosclerotic plaques of vehicle-treated and atorvastatin-treated mice. Data are presented as the mean ± SEM. ^∗∗∗^*P* < 0.001 vs. the control group alone.

To explore the molecular mechanism of atorvastatin inhibition of inflammation, we examined the protein levels of NLRP3 in aortic protein homogenate using western blotting. As shown in **Figures 3A,B**, atorvastatin significantly reduced the abundance of NLRP3 inflammasomes (*p* < 0.05). Moreover, carotid vulnerable plaques were stained for NLRP3. In **Figure 3C**, the NLRP3 protein level was significantly decreased by atorvastatin treatment, which suggested that atorvastatin suppresses inflammation by inhibiting the activation of NLRP3 inflammasomes.

### Atorvastatin Upregulated the Autophagy Level *in Vivo*

**FIGURE 4:**
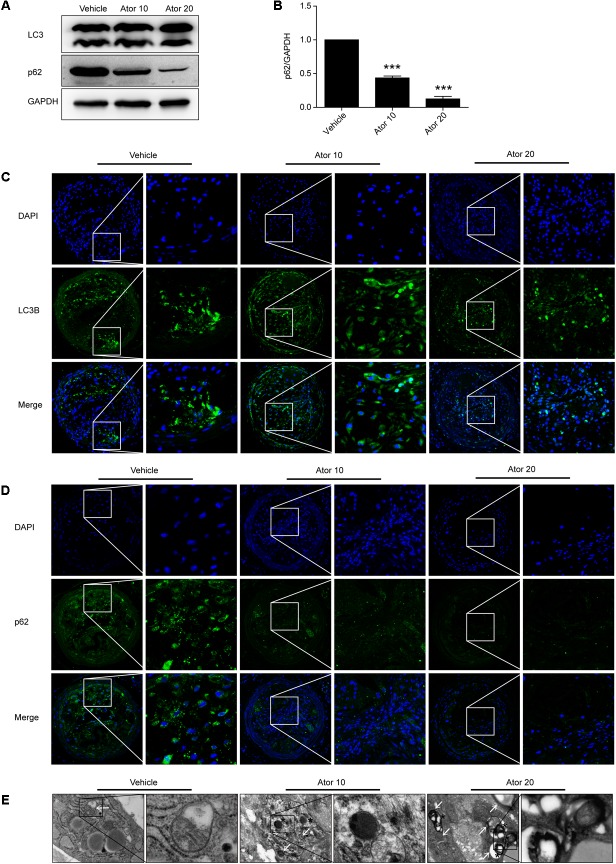
Atorvastatin enhanced autophagy in vulnerable atherosclerotic plaques in ApoE^-/-^ mice. **(A)** The expression of LC3 and SQSTM1/p62 were evaluated using western blotting in whole-aorta lysates from vehicle-treated and atorvastatin-treated mice, *n* = 3. **(B)** Quantitative analysis of SQSTM1/p62 are presented in histogram. **(C,D)** Immunofluorescence staining for LC3B and p62 in vulnerable atherosclerotic plaques. **(E)** Transmission electron microscopy was used to evaluate autophagy in vulnerable atherosclerotic plaques (magnification, 24500×). The white arrows represent autophagosomes; the asterisks represent autolysosomes and even represent myeloid structures. Values are expressed as the mean ± SEM of three independent experiments. ^∗∗∗^*P* < 0.001 vs. the control group alone.

We further investigated the detailed mechanism of atorvastatin’s effects on inflammation and the stability of atherosclerotic plaques. Previously, we showed that atorvastatin alleviated LPS-induced inflammation via upregulation of autophagy in RAW264.7 macrophages (Han et al., 2017). Therefore, we detected the effect of atorvastatin on autophagy *in vivo*. The expression of LC3II and SQSTM1/p62 in vulnerable atherosclerotic plaques were detected by western blotting and immunofluorescence staining for LC3B and p62. LC3B plays an important role in autophagosome formation, and an elevated p62 level is closely related to autophagy impairment. **Figures 4A,B** shows that atorvastatin treatment significantly decreased the level of SQSTM1/p62 in aortic plaques, indicating improved autophagy flux. Immunofluorescence analysis showed that the extent of positive staining for LC3B was significantly increased (**Figure 4C**), while the extent of positive p62 staining was significantly decreased in the atorvastatin treatment group (**Figure 4D**). Taken together, these results suggested that atorvastatin could enhance autophagy. We then employed TEM, the gold standard for the detection of autophagy, to assess the influence of atorvastatin on autophagy in atherosclerotic plaques. As shown in **Figure 4E**, autophagic vesicles were barely seen in the vehicle group; however, many autophagic vesicles and autolysosomes were observed in the atorvastatin10 group. In the atorvastatin20 group, we observed a number of myeloid structures (asterisks), which represent the residue after autolysosomes digestion. Taken together, these results suggested that atorvastatin attenuates inflammation and improves the stability of vulnerable plaques by upregulating autophagy *in vivo*. To verify this hypothesis, *in vitro* experiments were performed.

### Atorvastatin Restored Ox-LDL-Induced Impaired Autophagy Flux in RAW264.7 Cells

**FIGURE 5:**
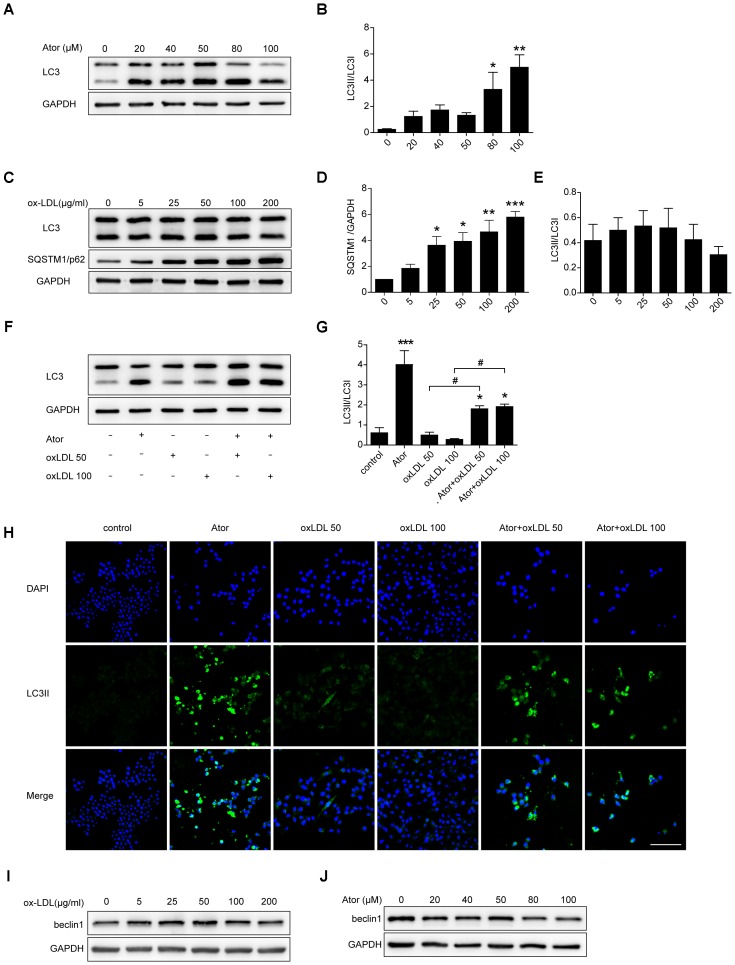
Atorvastatin restored impaired autophagy induced by ox-LDL in RAW264.7 cells. **(A)** LC3 expression in RAW264.7 cells treated with different concentrations of atorvastatin for 24 h. **(B)** Quantitative analysis of LC3II/LC3I, *n* = 3. **(C)** RAW264.7 cells were treated with different concentrations of ox-LDL (0, 5, 25, 50, 100, and 200 μg/ml) for 24 h. The expression of autophagy markers (LC3 and SQSTM1/p62) were detected by western blotting. **(D,E)** Histograms showing the quantitative analysis of LC3 and SQSTM1/p62, *n* = 3. ^∗^*P* < 0.05 vs. untreated cells, ^∗∗^*P* < 0.01 vs. untreated cells, ^∗∗∗^*P* < 0.001 vs. untreated cells. **(F)** An aliquot of RAW264.7 macrophage cells was pretreated with atorvastatin (100 μM) for 1 h, followed by stimulation with two concentrations of ox-LDL (50 or 100 μg/ml) for 24 h. The other aliquot of the macrophages was treated with atorvastatin (100 μM) or ox-LDL (50 or 100 μg/ml) alone for 24 h. The expression of LC3 was assessed by immunoblotting. **(G)** Densitometric measurement of the ratio of LC3II/LC3I from **(F)**, *n* = 4. **(H)** Cells were stimulated as prescribed above and immunofluorescence staining for LC3B was performed, scale bar = 200 μm. **(I)** Beclin1 expression in different concentrations of ox-LDL treatment cells. **(J)** Beclin1 expression in different concentrations of atorvastatin treatment cells. Data are presented as the mean ± SEM. ^∗^*P* < 0.05 vs. control cells, ^∗∗∗^*P* < 0.001 vs. control cells, ^#^*P* < 0.05 vs. the ox-LDL group alone. All cell experiments were repeated three times independently. Ator, atorvastatin 100 μM. Ator+ox-LDL 50, cells treated with 50 μg/ml ox-LDL in the presence of atorvastatin 100 μM. Ator+ox-LDL 100, cells treated with 100 μg/ml ox-LDL in the presence of atorvastatin 100 μM.

Ox-LDL plays a pivotal role in the initiation and progression of atherosclerosis; therefore, we explored the role of atorvastatin in ox-LDL-induced macrophages. As shown in **Figures 5A,B**, atorvastatin treatment could increase the ratio of LC3II/LC3I, indicating enhanced autophagy, which is consistent with our previous study. Several studies have illustrated that ox-LDL blocks autophagy flux. To eliminate the effect of ox-LDL and to further verify this effect of ox-LDL, we employed different concentrations of ox-LDL to stimulate RAW264.7 cells. The expression of SQSTM1/p62 increased markedly under stimulation by ox-LDL and peaked at an ox-LDL concentration of 200 μg/ml (**Figures 5C,D**). However, there were no obvious changes in the ratio of LC3II/LC3I (**Figures 5C,E**), although the ratio of LC3II/LC3I decreased slightly as the concentration of ox-LDL increased. Thus, the results strongly suggested that ox-LDL blocked autophagy flux in macrophages, and thus impaired autophagy. We then treated cells with two different concentrations of ox-LDL in the presence or absence of atorvastatin. Surprisingly, atorvastatin significantly upregulated the ratio of LC3II/LC3I in macrophages induced with ox-LDL, indicating that atorvastatin rescued ox-LDL-induced autophagy dysfunction (**Figures 5F,G**). In addition, immunofluorescence staining of LC3II, a marker of autophagic vesicle formation, significantly increased after treatment with atorvastatin compared with that in the control group, and atorvastatin significantly enhanced the level of LC3II compared with that in the two ox-LDL treatment groups (**Figure 5H**). These results suggested that atorvastatin could restore the ox-LDL-induced autophagy dysfunction in macrophages. In addition, we detected the protein expression of beclin1 from different treatment of RAW264.7 cells and we did not observe any obvious change in the expression of beclin1 (**Figures 5I,J**). The possible reason is that beclin1 is an important part of the highly conserved core complex which is composed of beclin1 and class III phosphatidylinositol 3-kinase (PI3K). The core complex is essential for the localization of autophagic proteins (such as Atg5 and Atg7) to the phagophore. Thus, beclin1 regulates the very initial step of autophagy activity and atorvastatin may regulate autophagy through non-canonical pathway.

### Atorvastatin Significantly Attenuated Ox-LDL-Induced Macrophage Lipid Accumulation and Inflammatory Cytokines Secretion by Inducing Autophagy

**FIGURE 6:**
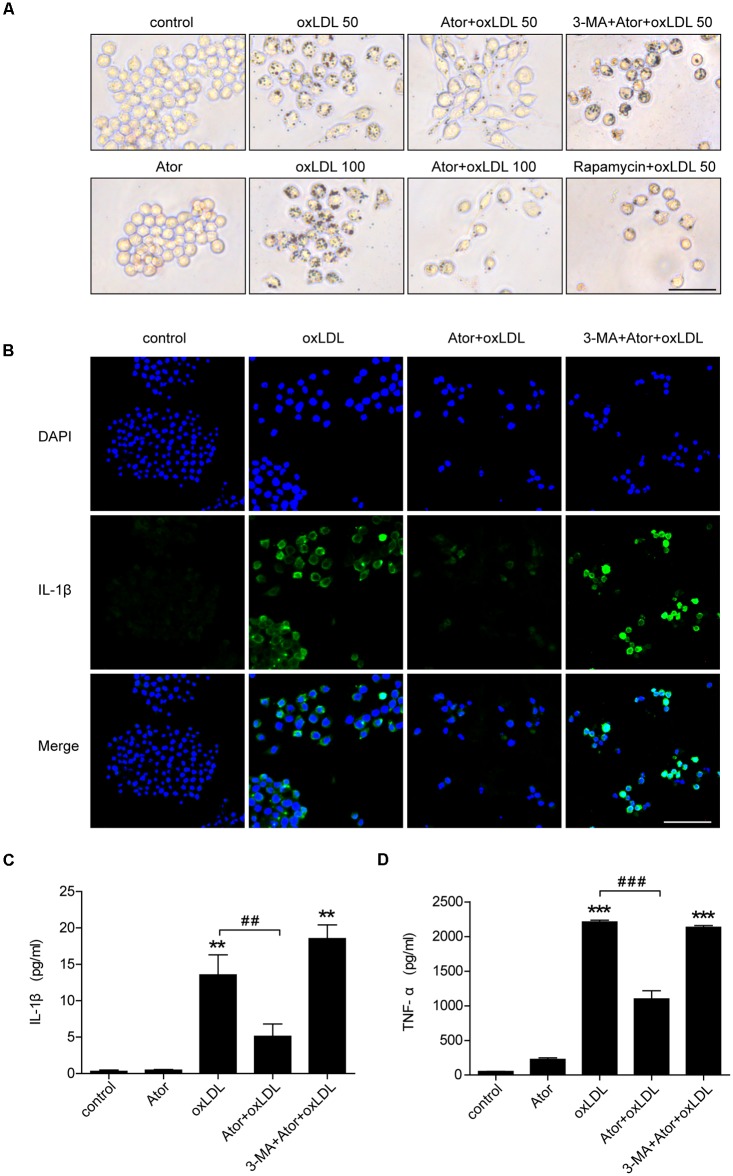
Atorvastatin decreased foam cell formation and suppressed inflammatory cytokines secretion induced by ox-LDL via enhancing autophagy in RAW264.7 cells. **(A)** Representative Oil Red O staining images of murine macrophages (magnification, ×200). Cells were treated with the vehicle solution (control), atorvastatin 100 μM (Ator), followed by treatment with ox-LDL 50 or 100 μg/ml, or combined with 3-MA (5 mM), or rapamycin (10 nM) for 24 h, respectively, scale bar = 25 μm. **(B)** Immunofluorescence staining for IL-1β in cells treated with control, ox-LDL (50 μg/ml), atorvastatin (100 μM), or with 3-MA (5 mM), scale bar = 50 μm. **(C,D)** Cells were treated with control, atorvastatin (100 μM), ox-LDL (50 μg/ml), or atorvastatin with ox-LDL (Ator+ox-LDL), or combined with 3-MA (5 mM), respectively, and the cell culture supernatants were collected. The secretion of IL-1β and TNF-α was assessed in the supernatants using ELISA, *n* = 3. Data are presented as mean ± SEM of at least three repeated experiments. ^∗∗^*P* < 0.01 vs. the control group alone, ^##^*P* < 0.01 vs. the ox-LDL group alone, ^∗∗∗^*p* < 0.001 vs. the control group, ^###^*p* < 0.001 vs. the ox-LDL group. 3-MA, 3-methyladenine.

To evaluate the effect of atorvastatin on ox-LDL-induced lipid deposition, we used ox-LDL to induce foam cell formation in RAW264.7 cells. As shown in **Figure 6A**, atorvastatin significantly attenuated ox-LDL-induced foam cell formation, as assessed by Oil Red O staining, and this effect could be abolished by 3-MA, a specific inhibitor of autophagy. By contrast, rapamycin, an autophagy inducer, also effectively ameliorated ox-LDL-induced lipid accumulation in RAW264.7 cells (**Figure 6A**). We further explored atorvastatin’s effect on inflammatory cytokines secretion in ox-LDL-induced macrophages. **Figure 6B** shows that atorvastatin could inhibit ox-LDL-induced IL-1β release, while 3-MA attenuated the suppressive effect of atorvastatin. As shown in **Figures 6C,D**, IL-1β and TNF-α secretion were suppressed by atorvastatin treatment compared with ox-LDL stimulation. However, 3-MA abolished the anti-inflammatory effect of atorvastatin. Therefore, we concluded that atorvastatin significantly attenuated foam cell formation and suppressed inflammation by inducing autophagy.

### Atorvastatin Upregulated Autophagy Through the mTOR Pathway and Inhibited the Activation of NLRP3 Inflammasome in RAW264.7 Cells

**FIGURE 7:**
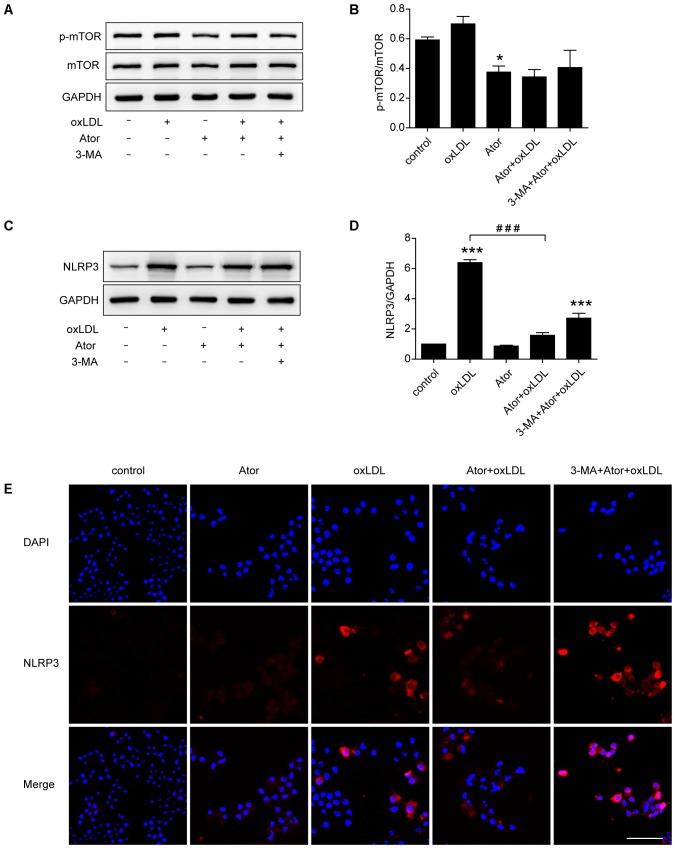
Atorvastatin inhibited the expression of NLRP3 and upregulated autophagy via the mTOR pathway. **(A)** Western blotting analysis of the levels of phospho-mTOR (p-mTOR) and total mTOR. **(B)** Quantitative analysis of the ratio of p-mTOR/mTOR, *n* = 3. **(C)** Western blotting analysis of the expression of NLRP3 in RAW264.7 cells after different treatments. **(D)** Histogram of the quantitative analysis of the data in **(C)**, after normalization to the GAPDH densitometric measurement, *n* = 3. **(E)** Immunofluorescence staining for NLRP3 in cells after different treatments; scale bar = 50 μm. Data are presented as the mean ± SEM. ^∗^*P* < 0.05 vs. the control, ^∗∗∗^*P* < 0.001 vs. the control, ^###^*P* < 0.0001 vs. the ox-LDL group alone. All cell experiments were repeated three times independently. Ator, atorvastatin 100 μM; ox-LDL, ox-LDL 50 μg/ml; 3-MA, 3-methyladenine 5 mM.

*In vivo* experiments confirmed that atorvastatin could inhibit the activation of NLRP3 inflammasomes and subsequent inflammation in atherosclerotic plaques. To verify the specific molecular mechanism of atorvastatin in regulating autophagy, western blotting analysis of mTOR and p-mTOR were carried out. The results showed that the ratio of p-mTOR/mTOR decreased after atorvastatin treatment compared with the control group (**Figures 7A,B**), indicating the involvement of the mTOR signaling pathway. As shown in **Figures 7C–E**, western blotting and immunostaining showed that atorvastatin significantly reduced the abundance of NLRP3 in ox-LDL-induced macrophages. However, the effect of atorvastatin on inhibiting activation of NLRP3 inflammasome was impeded by 3-MA treatment. These results suggested that atorvastatin could upregulate autophagy by inhibiting the phosphorylation of mTOR to inhibit the activation of NLRP3 inflammasomes.

### Atorvastatin Can Still Exert Anti-inflammatory and Attenuate Lipid Deposition Effects Under the Involvement of Chloroquine

**FIGURE 8:**
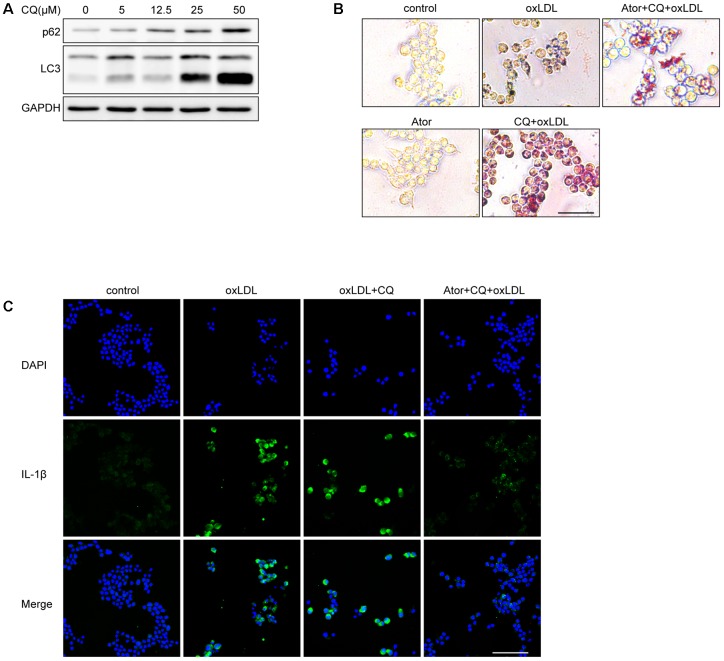
Atorvastatin can still exert anti-inflammatory and attenuate lipid deposition effects under the involvement of chloroquine. **(A)** The protein expression of LC3 and SQSTM1/p62 with different concentrations of chloroquine (CQ) treatment. **(B)** Representative Oil Red O staining images of murine macrophages. Cells were treated with the vehicle solution (control), ox-LDL 50 μg/ml, followed by treatment with chloroquine (25 μM), or atorvastatin 100 μM (Ator), followed by treatment with ox-LDL 50 μg/ml and chloroquine for 24 h, respectively, scale bar = 25 μm. **(C)** Immunofluorescence staining for IL-1β of cells treated with control, ox-LDL (50 μg/ml), atorvastatin (100 μM), or with chloroquine (25 μM), scale bar = 50 μm.

We used different concentrations of chloroquine (CQ) to stimulate RAW264.7 cells and it turned out CQ increased the ratio of LC3II/LC3I and SQSTM1/p62, indicating that the autophagy flux was impeded (**Figure 8A**). According to **Figure 8A**, we used 25 μM as our experimental concentration. Oil Red O staining revealed that CQ even exacerbated the lipid accumulation induced by ox-LDL (**Figure 8B**). While, with the atorvastatin administration, the lipid accumulation was significantly alleviated. Moreover, we found that atorvastatin could inhibit IL-1β expression with CQ involved (**Figure 8C**). In conclusion, we speculate that atorvastatin could restore the impaired autophagy flux impeded by CQ.

### The Effect of Atorvastatin on Apoptosis

**FIGURE 9:**
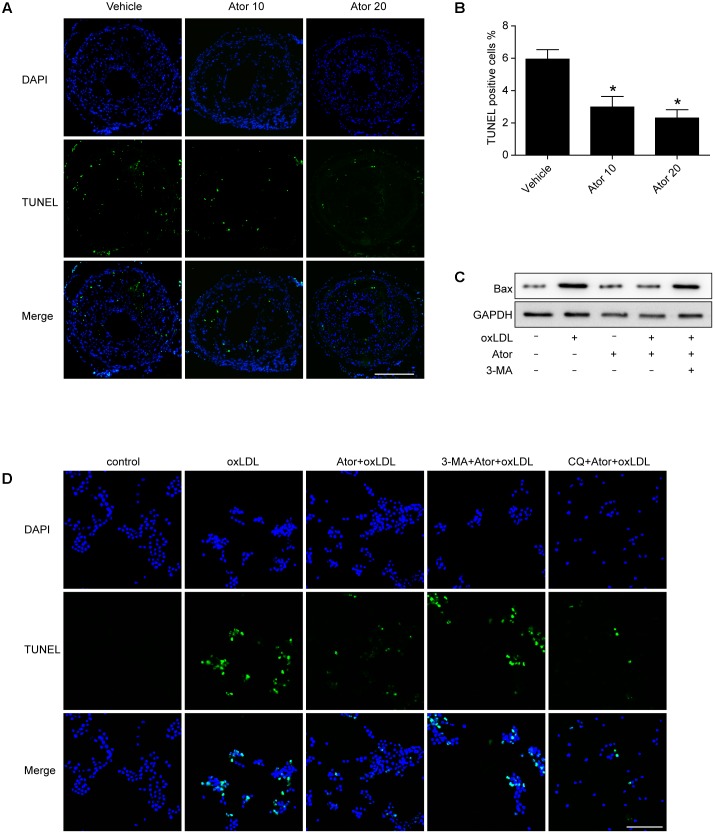
Atorvastatin inhibited apoptosis both *in vivo* and *in vitro*. **(A)** Representative TUNEL staining of vulnerable atherosclerotic plaques. Scale bar = 200 μm. **(B)** Quantitative analysis of TUNEL positive rate. *n* = 3, ^∗^*P* < 0.05 vs. vehicle. **(C)** The expression of Bax was evaluated with western blot. Ator, atorvastatin 100 μM; ox-LDL, ox-LDL 50 μg/ml; 3-MA, 3-methyladenine 5 mM. **(D)** Representative TUNEL staining from different treatment of RAW264.7 cells. CQ, chloroquine 25 μM. Scale bar = 50 μm.

In order to detect whether atorvastatin could inhibit apoptosis, we conducted TUNEL assays in vulnerable atherosclerotic plaques. The results show that atorvastatin treatment significantly reduced the TUNEL positive rate *in vivo*. The apoptosis marker, Bax, was also detected in RAW264.7 cells. The western blot results showed that atorvastatin could also inhibit the Bax expression induced by ox-LDL. TUNEL analysis was also conducted in RAW264.7 cells with different treatments. Consistent with the experiment *in vivo*, atorvastatin could inhibit apoptosis. Therefore, the above results demonstrated that autophagy induced by atorvastatin is concomitant with decreased apoptosis (**Figure 9**). Interestingly, with the 3-MA treatment, the anti-apoptosis effect of atorvastatin was offset, while CQ did not affect the anti-apoptosis effect (**Figure 9D**). The possible reason may be that CQ impeded the fusion of autophagosomes and lysosomes, while 3-MA inhibited the very beginning stage of autophagy activation. From the results we got, atorvastatin could restore the autophagy flux so that CQ could not inhibit the anti-apoptosis effect of atorvastatin.

**FIGURE 10:**
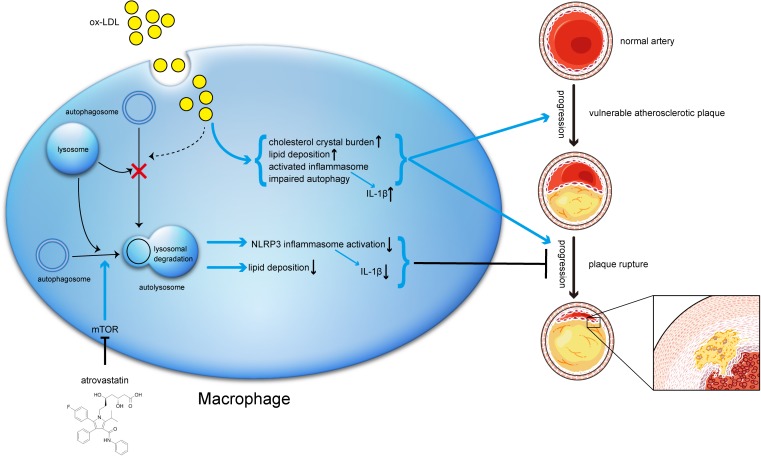
Schematic description of the effects of atorvastatin on autophagy, and the relationship between autophagy, inflammation, and atherosclerosis progression. During the early stage of atherosclerosis, macrophage autophagy is intact and exerts its normal effects. However, when exposed to excessive ox-LDL, autophagy flux is blocked through mechanisms that might involve cholesterol crystal overload or lysosomal leakage. Impaired autophagy results in lipid accumulation and activated inflammasomes, both of which in turn exacerbate atherosclerosis. Meanwhile, atorvastatin could upregulate autophagic activity through the mTOR pathway to inhibit NLRP3 inflammasome activation and alleviate lipid deposition, subsequently mitigate inflammation and stabilizing vulnerable atherosclerotic plaques.

## Discussion

In the present study, we demonstrated that atorvastatin decreased the vulnerability of advanced atherosclerotic plaques, and inhibited the inflammatory response and the activation of NLRP3 inflammasomes, by inducing autophagy in ApoE^-/-^ mice. We also observed that atorvastatin attenuated foam cell formation, suppressed inflammatory cytokines secretion, and upregulated autophagy in RAW264.7 macrophage cells stimulated with ox-LDL. Furthermore, the effects of atorvastatin involve the inhibition of mTOR phosphorylation and NLRP3 inflammasome formation. Thus, we concluded that atorvastatin exerted an anti-inflammatory effect, attenuated lipid deposition, and improved the stability of vulnerable atherosclerotic plaques by modulating autophagy.

Atherosclerosis is the main pathophysiological basis of acute coronary syndrome, myocardial infarction, and stroke, and has become the leading cause of death and disability worldwide. The rupture of vulnerable atherosclerotic plaques is the primary cause of coronary thrombosis and subsequent myocardial infarction.

Statins comprise HMG-CoA reductases and have long been used routinely in patients with atherosclerosis because of their lipid lowering effect. Recently, statins have shown other potential effects in the treatment of cardiovascular disease, such as inhibiting inflammation (Parikh et al., 2010; Toepfer et al., 2011; Antonopoulos et al., 2012; Yang et al., 2012; Wang et al., 2014; Gao et al., 2016). Statins have also been shown to attenuate plaque vulnerability by downregulating the expression of EMMPRIN (extracellular matrix metalloproteinase inducer) and certain chemokines (Nie et al., 2014; Liang et al., 2017). In the present study, we showed that atorvastatin inhibited the atherosclerotic lesions formation and improved the stability of vulnerable atherosclerotic plaques.

Accumulating evidence has confirmed the ability of atorvastatin to inhibit the secretion of IL-1β and TNF-α induced by LPS (Pun et al., 2015; Han et al., 2017). Our previous studies demonstrated that atorvastatin suppressed inflammatory responses via inhibition of extracellular signal-regulated kinase (ERK) phosphorylation and cyclooxygenase-2 (COX-2) expression in macrophages induced by ox-LDL (Shao et al., 2012) and exerted anti-inflammatory effects by suppressing the secretion of IL-1β and TNF-α induced with LPS in macrophages (Han et al., 2017). Consistent with these previous studies, the results presented here also showed that atorvastatin markedly inhibited IL-1β and TNF-α secretion from macrophages exposed to ox-LDL. Moreover, atorvastatin treatment ameliorated the accumulation of lipid droplets in macrophages exposed to ox-LDL.

Furthermore, *in vivo*, atorvastatin treatment significantly decreased the level of certain inflammatory factors, such as IL-1β, TNF-α, and IL-18. In particular, in vulnerable atherosclerotic plaques and in macrophages activated by ox-LDL, atorvastatin treatment significantly decreased the expression of IL-1β. Thus, atorvastatin inhibited the inflammatory response, reduced lipid deposition, and improved the stability of vulnerable atherosclerotic plaques.

Autophagy is a highly evolutionarily conserved process that is responsible for quality control of proteins and other cytobiological processes. Except for the fundamental role of regulating cell death and survival, accumulating evidence suggests that autophagy participates in various physiological activities, and loss of autophagy leads to many pathological conditions. Autophagy is active in atherosclerotic plaques (Magne et al., 2015) and plays a protective role in advanced atherosclerotic plaques (Liao et al., 2012). Upregulating autophagy using adiponectin exerted an atheroprotective effect (Li et al., 2015). We hypothesized a possible interaction between the atorvastatin-regulated inflammatory response and the stability of atherosclerotic plaques via modulation of autophagy.

Among the autophagy-related proteins, the conversion of LC3-I to LC3-II is widely used as a marker of autophagy activation. SQSTM1, also known as p62, is responsible for recognizing and transporting cargoes that need to be degraded to autophagy vesicles and is degraded along with the cargo. Thus, the level of p62 is negatively correlated with the level of autophagy flux, which means that an elevated p62 level probably indicates impeded autophagy flux. In the present study, we showed that the LC3B ratio was significantly increased and p62 staining was significantly decreased in the atorvastatin treatment groups. TEM showed abundant autophagic vesicles and autolysosomes. In addition, atorvastatin increased the ratio of LC3II/LC3I and the positive staining of LC3B in ox-LDL stimulated macrophages, which indicated that atorvastatin upregulated autophagy. However, atorvastatin didn’t affect the expression of beclin1 in macrophages (**Figure 5J**) and the possible reason maybe that beclin1 regulates the very initial step of autophagy activity and atorvastatin may regulate autophagy through non-canonical way. The protective effects of atorvastatin were inhibited by the autophagy inhibitor 3-MA, indicating the involvement of autophagy in the anti-inflammatory, atheroprotective, and lipid deposition lowering properties of atorvastatin.

Autophagy defects contribute to inflammasomes activation and subsequent exacerbation of atherosclerosis, whose underlying mechanisms might be the accumulation of lipid and cholesterol crystals in lysosomes, resulting in the instability of the lysosomal membrane and the destruction of the integrity of the lysosomal membrane in activated inflammasomes (Sergin and Razani, 2014).

The NLRP3 inflammasome is closely associated with the secretion of IL-1β. Inflammasomes, which are activated by cell infection and stress, have the potential to trigger the activation of caspase-1, which cleaves the pro-IL-1β and IL-18 into their mature forms, resulting in IL-1β and IL-18 secretion (Peng et al., 2015). The assembled inflammasomes them undergo ubiquitination and are recruited by p62, leading to their transport to autophagosomes. Inflammasomes are tightly associated with atherosclerosis. Silencing of NLRP3 impeded atherosclerosis progression and stabilized atherosclerotic plaques (Zheng et al., 2014). Activation of NLRP3 inflammasomes increased lipid deposition and promoted atherosclerosis progression (Li et al., 2014). In the present study, we found that atorvastatin suppressed NLRP3 inflammasome activation in vulnerable atherosclerotic plaques and in activated macrophages stimulated by ox-LDL.

Intact VSMC function is crucial in protecting the vessel wall against atherosclerosis and VSMC senescence promotes atherosclerosis and plaque vulnerability (Wang J. et al., 2015). In response to oxidative stress-inducing stimuli, which prevail in atherosclerotic lesions (Wang and Bennett, 2012), the VSMC has mainly three choices, either fight, adapt or die, basically through autophagy, senescence or apoptosis (Grootaert et al., 2018). Recently, the interesting link between VSMC senescence and autophagy has been uncovered, consolidates the general consensus that successful autophagy promotes VSMC survival. Defective autophagy in VSMCs (atg7^-/-^ VSMCs) accelerates senescence and promotes ligation-induced neointima formation and diet-induced atherogenesis, as shown by increased total collagen deposition, nuclear hypertrophy, CDKN2A upregulation, and GLB1 activity (Grootaert et al., 2015). In contrast, rapamycin exerts anti-senescence effects in VSMCs via inhibition of the mTOR pathway (Tan et al., 2016), which is known to be overactivated in senescent cells (Luo et al., 2017). As in the vessel calcification, atorvastatin protects VSMCs from TGF-β1-stimulated calcification via autophagy activation (Liu et al., 2014). Moreover, statins could prevent premature aging, leading to enhanced telomere protection through upregulating TRF2 (Spyridopoulos et al., 2004). Interestingly, senescent human VSMCs release high levels of multiple cytokines and chemokines through an IL-1α-dependent SASP (senescence-associated secretory phenotype), activating multiple inflammasome components (such as NLRP3, caspase-1, IL-1β), and priming adjacent cells to a pro-inflammatory state (Gardner et al., 2015). Considering our observations that atorvastatin could regulate autophagy and inhibit the inflammasome activation, we speculate that atorvastatin could slow senescence and inhibit apoptosis through activating autophagy, which might be a promising therapeutic target in the treatment of atherosclerosis.

Studies have demonstrated that atorvastatin plays a positive role in myocardial hypertrophy by augmenting autophagy through the Akt/mTOR pathway (Wang W. et al., 2015). Toepfer et al. (2011) also showed that atorvastatin activated *LC3II* transcription in prostate cancer PC3 cells by modulating the ERK and JUN N-terminal kinase (JNK) pathways. In our study, cells incubated with atorvastatin showed lower levels of mTOR phosphorylation, which indicated the involvement of the mTOR pathway in the anti-atherosclerotic effects of atorvastatin.

There is controversy surrounding the effects of ox-LDL on autophagy, with most opinions stating that it obstructs autophagy flux, whereas some researchers insist that ox-LDL activates autophagy. Our results showed that although stimulation with ox-LDL did not significantly affect the ratio of LC3II/LC3I, it did increase the level of p62, which suggested blockage of autophagy flux. We concluded that ox-LDL blocked autophagy in an as-yet undiscovered manner. Ox-LDL, cholesterol crystals, or other unknown substances, such as ceroid, can cause lysosomal membrane instability in vulnerable plaques, leading to lysosome content leakage. Consequently, autophagosomes and lysosomes cannot fuse, eventually leading to the blockage of autophagy flux (Duewell et al., 2010; Schrijvers et al., 2011). Macrophages isolated from atherosclerotic plaques also displayed features of lysosomal dysfunction. P62-deficient mice are prone to develop more advanced atherosclerotic plaques and p62-specific knockout macrophages are more likely to undergo apoptosis and produce more IL-1β (Sergin et al., 2016). Thus, as plaques evolve into vulnerable atherosclerotic plaques, their autophagy flux appears to be impaired (Schrijvers et al., 2005; Sergin and Razani, 2014). Therefore, we hypothesized that repairing dysfunctional autophagy could stabilize vulnerable atherosclerotic plaques.

In support of this hypothesis, we used CQ, which could block the autophagy flux, to explore whether atorvastatin could still exert its effects. The results suggested that atorvastatin could attenuate lipid deposition and inhibit IL-1β expression (**Figure 8**). Above all, we determined that atorvastatin significantly decreased the plaque burden, reduced the vulnerability of plaques, mitigated the inflammatory response, inhibited inflammasome activation, and attenuated lipid deposition by enhancing autophagy. In addition, we also verified that atorvastatin had the effect of inhibiting apoptosis both *in vivo* and *in vitro* (**Figure 9**). However, the regulation of autophagy is very complex and the details have not been determined definitively. Although autophagy exerts anti-atherogenic properties, the expectation that activating autophagy will inhibit atherosclerosis has not made much headway in the short term because all known drugs with autophagy-enhancing capabilities have obvious side effects, for example, rapamycin can cause hyperlipemia and immunosuppression. Moreover, the Toll-like receptor 7 (TLR7) ligand is associated with an elevated inflammatory response. Our findings may provide new insights into the molecular mechanism of atorvastatin and its novel therapeutic role in the treatment of atherosclerosis. The proposed mechanism of these effects is summarized in **Figure 10**. In the near future, regulating autophagy might develop into a promising strategy to stabilize atherosclerotic plaques and thus ameliorate atherosclerotic cardiovascular diseases.

## Ethics Statement

All animal experiments were approved by the Institutional Animal Care and Use Committee of Renji Hospital.

## Author Contributions

QS conceived and designed the research. SP, X-YC, and Q-QX performed the experiments. SP and L-WX analyzed the data. JP and BH contributed reagents, materials, and analysis tools. All authors read and approved the final version of the manuscript.

## Conflict of Interest Statement

The authors declare that the research was conducted in the absence of any commercial or financial relationships that could be construed as a potential conflict of interest.
