# Supplementary material for: Atorvastatin Inhibits Inflammatory Response, Attenuates Lipid Deposition, and Improves the Stability of Vulnerable Atherosclerotic Plaques by Modulating Autophagy
Source: Front Pharmacol. 2018 May 3;9:438. doi: 10.3389/fphar.2018.00438 (PMC5943597; doi:10.3389/fphar.2018.00438)
Supplement: Supplementary file 1 [file Presentation_1.PDF]

Supplementary Materials

Terminal Deoxynucleotidyl Transferase-mediated dUTP-biotin nick end labeling (TUNEL) Assay

Apoptotic cells were detected by TUNEL staining, using the In Situ Cell Death Detection Kit, Fluorescein (catalog number 11684795910; Roche Applied Science, Mannheim, Germany), following the manufacturer's instructions. In brief, sections or RAW264.7 cells underwent different treatments were rinsed with PBS and fixed in 4% paraformaldehyde for 1h at room temperature, then washed with PBS, and permeabilized with freshly prepared 0.1% Triton X-100 and 0.1% sodium citrate for 10 min on ice. After washing with PBS, the coverslips were overlaid with 50 μl of TUNEL reaction mixture, according to the manufacturer's instructions, and incubated for 1 h at 37 ℃. Finally, cells were washed in PBS, and coverslips were mounted onto slides with antifade mountant with DAPI (P36965, ProLong Diamond Antifade Mountant, Life Technologies, Grand Island, NY, USA). Cells were imaged using a fluorescence microscope (Leica DM3000B, Germany). The percentage of TUNEL-positive nuclei (green) was quantified using an ImageJ software.

Supplementary Figure.1

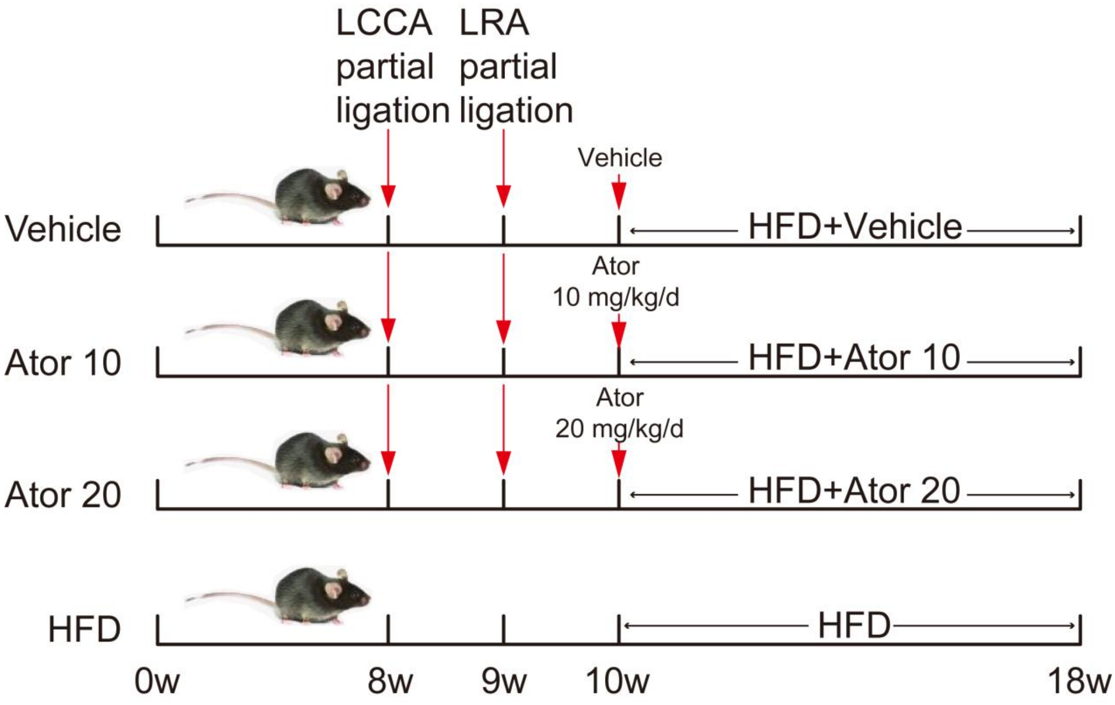

Supplementary Figure.1 Mice grouping pattern. ApoE$^{-/-}$ mice were divided into four groups: HFD (mice without surgery), Vehicle (saline solution), Ator 10 (atorvastatin 10 mg/kg/day), Ator 20 (atorvastatin 10 mg/kg/day). HFD group means that mice received only a high-fat-diet without partial ligation surgery, while the other three groups not only received a high-fat-diet but also underwent the partial ligation of left common carotid artery and left renal artery. Vehicle group means that mice were lavaged with normal saline. HFD, high fat diet. LCCA, left common carotid artery. LRA, left renal artery.
